# Early Brain Damage Affects Body Schema and Person Perception Abilities in Children and Adolescents with Spastic Diplegia

**DOI:** 10.1155/2019/1678984

**Published:** 2019-08-18

**Authors:** Niccolò Butti, Rosario Montirosso, Lorenzo Giusti, Luigi Piccinini, Renato Borgatti, Cosimo Urgesi

**Affiliations:** ^1^Scientific Institute, IRCCS E. Medea, Neuropsychiatry and Neurorehabilitation Unit, Bosisio Parini, Lecco, Italy; ^2^Scientific Institute, IRCCS E. Medea, 0-3 Centre for the At-Risk Infant, Bosisio Parini, Lecco, Italy; ^3^Scientific Institute, IRCCS E. Medea, Specialist Functional Rehabilitation Unit, Bosisio Parini, Lecco, Italy; ^4^Laboratory of Cognitive Neuroscience, Department of Languages and Literatures, Communication, Education and Society, University of Udine, Italy

## Abstract

Early brain damage leading to cerebral palsy is associated to core motor impairments and also affects cognitive and social abilities. In particular, previous studies have documented specific alterations of perceptual body processing and motor cognition that are associated to unilateral motor deficits in hemiplegic patients. However, little is known about spastic diplegia (SpD), which is characterized by motorial deficits involving both sides of the body and is often associated to visuospatial, attentional, and social perception impairments. Here, we compared the performance of a sample of 30 children and adolescents with SpD (aged 7-18 years) and of a group of age-matched controls with typical development (TD) at two different tasks tapping on body representations. In the first task, we tested visual and motor imagery abilities as assessed, respectively, by the object-based mental rotation of letters and by the first-person transformations for whole-body stimuli. In the second task, we administered an inversion effect/composite illusion task to evaluate the use of configural/holistic processing of others' body. Additionally, we assessed social perception abilities in the SpD sample using the NEPSY-II battery. In line with previously reported visuospatial deficits, a general mental imagery impairment was found in SpD patients when they were engaged in both object-centered and first-person mental transformations. Nevertheless, a specific deficit in operating an own-body transformation emerged. As concerns body perception, while more basic configural processing (i.e., inversion effect) was spared, no evidence for holistic (i.e., composite illusion) body processing was found in the SpD group. NEPSY-II assessment revealed that SpD children were impaired in both the theory of mind and affect recognition subtests. Overall, these findings suggested that early brain lesions and biased embodied experience could affect higher-level motor cognition and perceptual body processing, thus pointing to a strict link between motor deficits, body schema alterations, and person processing difficulties.

## 1. Introduction

Cerebral palsy (CP) is a composite group of nonprogressive movements and postures' disorders, which results from an early brain lesion [1]. While the classification of CP is strictly focused on core motor symptoms, this condition is often accompanied by cognitive delay, neuropsychological impairments, and social behavior disorders [2–4]. In particular, spastic diplegia (SpD), which is characterized by motorial diseases involving both sides with a greater impairment to the lower limbs [5], has been primary associated with neuropsychological impairments in the visuoperceptual domain [6, 7]. Moreover, not only are SpD children compromised in basic visuospatial [8] and sensory-motor functions [9, 10] but they also show deficits in higher-level attention and executive functions [11–13]. These primary neuropsychological impairments could limit children with SpD to participate in social contexts, explaining, at least partially, their social difficulties [14]. Nevertheless, the social difficulties of children with SpD may be independent by other neuropsychological deficits [11] and may reflect alterations of areas involved in social perception [15].

At the neural level, SpD is often due to periventricular leukomalacia (PVL), an early damage involving the white matter bordering the lateral ventricles, the corticospinal tracts, and the optic radiations [11, 16–19]. Importantly, the extension of the white matter damage to parietooccipital regions of both hemispheres and to the right temporal lobe areas has been found to be related to the severity of deficits in the perception and understanding of others' actions [15]. Indeed, these areas are crucially involved in the visual processing of biological motion [20], which has been considered as a hallmark of social cognition [21].

Furthermore, perception and representation of our own body, based on motor and proprioceptive experience, is crucial not only in self-representation [22, 23] but also in social interactions [24]. Indeed, several studies have documented an overlap between the neurocognitive processes involved during action execution and action observation [25–27]. This shared activation allows us to map other people's actions into proprioceptive experiences, ultimately facilitating our understanding of others' motor, sensorial, emotional, and even cognitive states through embodiment [28–30]. Accordingly, our bodily states and experiences may influence our perception and representation of others' postures and movements, which in turn gives us information about the underlying emotional and cognitive meanings in social interactions [31–33]. Since CP patients could present impaired mapping between visual and proprioceptive information [34] and they experience their body and the world in an unstable perception-movement system [4], they may present specific social perception deficits.

Deficits in body and motor representations in children with CP have been widely investigated using the motor imagery task [35–38]. For instance, Frassinetti et al. [39] documented greater impairments in own or others' body perception in hemiplegic children with right or left hemisphere lesions, respectively, confirming their results in adult patients with unilateral brain lesion [40]. In an fMRI study, Chinier et al. documented specific patterns of brain responses in children with unilateral CP, highlighting that left brain damage affected the execution of motor imagery tasks more than right brain lesions [35]. In a similar vein, Jongsma et al. reported impaired performance for the affected contralesional hand in a hand laterality judgment task [37]. Importantly, motor imagery is underpinned by the same cognitive motor processes involved in action observation and motor planning [41] and it has been proposed as an innovative tool for motor rehabilitation [42, 43]. Further, motor imagery may be critically involved in high-level cognition [44] and a link between motor imagery, perception of biological movement, and social cognition has been proposed [15, 45].

Notably, most studies have focused primarily on motor imagery in hemiplegic patients [36, 46, 47], while only recently has research started to investigate body representation in individuals with SpD [48, 49]. Furthermore, the body representation impairments of CP children may extend beyond motor imagery and affect perceptual, semantic, and motor levels of body representation [50, 51]. Importantly, exploring how bilateral congenital lesions could affect own and others' body perception is important not only to study how SpD patients may represent their self and participate in social contexts but also to explore specific interventions and rehabilitation strategies [43, 52].

In line with previous researches on motor imagery [47, 53–55], we investigated the ability of participants in executing visuospatial mental transformations. In details, children and adolescents were required to perform left-right hand judgments on whole-body stimuli presented either in back view (accordingly to the participants' perspective) or in front view condition (thus, involving an own body rotation to align with the position of the stimulus). While in the first condition, participants could answer referencing to their body position, in the latter one, they had to perform a *first-person mental transformation*, which required the use of motor imagery. Further, participants were asked to perform a left-right side judgment task with a nonsocial stimulus, which was the letter F, presented in its canonical position or turned at 180° around the vertical axis. This latter condition required the use of visual imagery abilities to perform an *object-based mental transformation*. Although mental transformations for bodies and objects require comparable cognitive efforts, as revealed by similar performance since 8 years of age [55], they involve partially distinct cognitive processes and neural mechanisms [56, 57]. Thus, the comparison between the performance in executing mental rotation on bodies and letters allowed us to understand whether SpD children were selectively impaired in motor as compared to visual imagery abilities and whether they used different strategies for social and nonsocial stimuli.

In order to explore how deficits in self-body representations could affect the perception of others' body, a *visual body recognition task* was administered to participants. This task, based on both the inversion [58–63] and the composite illusion [64–67] effects, was designed to test the use of configural and holistic processing in perceiving body stimuli. Indeed, configural processing uses a dynamic template representation of the relations among different parts of the stimuli, in the context of the whole stimulus configuration, to perceive relevant social stimuli [68–70]. This perceptual processing strategy, which develops from repeated exposure to species-specific social stimuli [71, 72], is much more efficient than the detail-based processing that is involved in object perception [73]. Since the presentation of inverted stimuli disrupts it, a better performance is expected for upright as compared to inverted stimuli (so called: inversion effect). Thus, the inversion effect has been used as an index of configural processing in perceiving body stimuli [60, 74].

While configural processing consists in the detection of first-order and second-order relations among features, holistic processing refers to the perception of a social stimulus as a whole and not as a combination of single features [68]. Indeed, it represents a more refined perceptual ability tailored to specific social stimuli such as bodies and faces, facilitating identity and emotion recognition [75, 76]. Holistic processing is revealed by the composite illusion effect [66, 77, 78]; two identical top halves of bodies or faces combined with two different bottom halves are perceived as being different when they are aligned (with respect to vertical axis), but not when they are misaligned [79]. Since the presentation of misaligned stimuli disrupts the holistic processing, in a delayed same-different judgment focused on the top halves of the stimuli, a better performance is expected when the top and the bottom parts are misaligned rather than aligned [65–67].

According to the role of these perceptual processes in social interactions, alterations of configural and holistic processing have been found in pathologies with cognitive and social deficits, such as bulimia and anorexia nervosa [61, 80], schizophrenia [33, 81], autism spectrum [82], prosopagnosia [83, 84], and other acquired brain damage [40, 85].

The performance of pediatric patients with SpD aged 7-18 years was compared to that of age-matched controls. In line with data on hemiplegic individuals [50, 51], we expected that SpD children were impaired at different levels of body representation. Indeed, the cognitive-neuropsychological model of body representation proposed for adults [86] and then adapted for the evaluation of children with cerebral palsy [50] describes at least three distinct levels of body representation: body schema, body structural description, and body image. Here, we assessed body schema through the visuospatial imagery task and body structural description, namely, the category-specific representation that supports visual body perception, through the visual body recognition task. On the one hand, patients with SpD were expected to show general difficulties in operating mental transformations, in keeping with the core impairment of visuospatial abilities in SpD [6, 7, 87]. On the other hand, a specific deficit was expected for the bodily stimuli (i.e., *first-person mental transformations*) in SpD due to the primarily biased experience of their own body. Beside this deficit in motor imagery, we expected that patients with SpD could be affected also at a perceptual level of body representation. Since it has been demonstrated that embodied experience exerts an effect on body perceptual processing independently by visual expertise [88], SpD patients should show less effect of body inversion and/or composite illusion, reflecting limited use of configural and/or holistic body processing.

## 2. Materials and Method

### 2.1. Participants

Participants were children and adolescents with a diagnosis of spastic diplegia, referred to the Specialist Functional Rehabilitation Unit of the Scientific Institute IRCCS E. Medea (Bosisio Parini, Italy) for routine clinical and functional evaluation and for rehabilitation treatments. The inclusion criteria were age > 7 and <18 years; no severe or profound intellectual disability (full-scale intelligent quotient (FSIQ ≥ 35) [89]); no severe fine motor (Manual Ability Classification System (MACS ≤ 3) [9]), gross motor (Gross Motor Function Classification System (GMFCS ≤ 4) [90]), or visual impairments (visual acuity ≤ 6/12 [91]) that could interfere with the execution of the tasks. It is worth noting that 6 children and adolescents with SpD presented with Cortical Visual Impairment (CVI) [92]. However, all children with visual deficits (myopia, astigmatism, and hypermetropia) were equipped with corrective lenses (corrected vision for each eye > 8 for all patients), limiting the likely impact of visual impairments on performing the tasks. Furthermore, all recruited children did not present hearing impairments. Clinical information is further described in Table 1 according to the recent indications for classification of CP [5].

Eligible participants were identified by the attending physician and reported to the researchers. A research assistant approached the parents providing full information about the study. Parents were asked to sign a written informed consent before enrolling the children and adolescents. The study was approved by the Ethics Committee of the Scientific Institute IRCCS Eugenio Medea and by the Regional Ethics Committee of Friuli Venezia Giulia; procedures were in accordance to the 1975 Declaration of Helsinki. For each participant, a chart review was conducted in order to collect the following demographic and clinical measures: gender, age, clinical data, scores at the Manual Ability Classification System (MACS) [9] and at the Gross Motor Function Classification System (GMFCS) [90, 93], and full-scale intelligent quotient, (FSIQ) scores [94]. Thirty patients were recruited in the study, with greater prevalence of males (*n* = 26) than females (*n* = 4), in keeping with previous studies [95] and with the particular vulnerability of male preterm infants to early white matter damage [96].

SpD patients were compared to a group of 30 children and adolescents (16 girls) with typical development (TD) recruited from local schools and matched for age (*t*_58_ = −0.422; *p* = 0.675). As for the SpD group, parents of TD children were given with all information about the study and asked to sign a written informed consent. Demographic information for both groups and scores at the neuropsychological evaluation for the SpD group are reported in Table 2.

### 2.2. General Procedure

**Figure 1:**
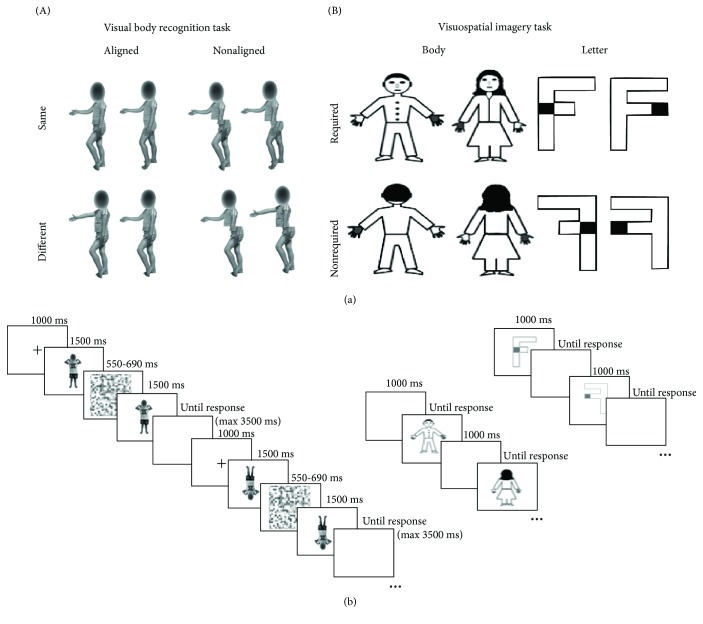
Structure of the tasks. (a) Illustration of the stimuli for the visual body recognition task (A) and the visuospatial imagery task (B). (b) Schematic representation of the timeline of the trials in the two tasks.

Clinical information is collected through a chart review. Functional motor abilities and, in particular, fine and gross motor functions were evaluated by the attending physician according to the most common classifications, i.e., MACS [9] and GMFCS [90]. Ophthalmologic and hearing impairments were assessed by specialist physicians as part of routine clinical evaluation as suggested by international guidelines for children with cerebral palsy [5, 97]. In a similar vein, we collected the most recent available FSIQ assessed with the Wechsler Intelligence Scale for children 4th edition (WISC-IV) [94]. For the experimental procedures, we followed the methods of Corti et al. [98]. Participants were tested in a silent room and were administered two subtests of the *Developmental Neuropsychological Assessment*—*2^nd^ Edition* (NEPSY-II) [99, 100] and the two experimental tasks, namely, the *visuospatial imagery task* and the *visual body recognition task*, in two different days. To administer the experiment, a 15.4-inch LCD monitor (resolution, 1024 × 768 pixels; refresh frequency, 60 Hz) was used, while stimulus presentation timing and randomization were controlled by using the E-prime 2 software package (Psychology Software Tools; http://www.pstnet.com/eprime) running on a PC. Participants were seated at a distance of approximately 60 cm from the computer screen and had to respond using the left or right button of the mouse. The order of task administration was counterbalanced across participants in each group. A detailed description of each experimental task is provided in Figure 1.

### 2.3. Visuospatial Imagery Task

The visuospatial imagery task required participants to perform right-left judgments on social-related stimuli, namely, a female-like or a male-like body manikin, and on nonsocial objects, namely, two differently written “F” letters (i.e., the lower arm could have the same or smaller length than the upper arm). The two types of stimuli were presented in separate blocks, and the participants were asked a laterality judgement task. In particular, the body drawings had one hand marked in grey and participants were asked to judge whether the marked hand was the right or the left one, according to the manikin's perspective. The body drawing stimuli could be shown in a front view or in a back view condition. In this latter condition, the manikin was presented in accord with the children's perspective, so that they could answer without any mental transformation. Conversely, the front-view stimuli required a first- to third-person perspective transformation to be responded. Regarding the nonsocial object, the letter F was presented with a grey square on one side and participants were asked to judge if the square was on the left or the right side of the standard letter. The letter F could be presented in the canonical position (unturned condition) or rotated at 180° around its vertical axis (turned condition). Only in the turned condition had the participants to operate a mental transformation of the object to respond. Thus, both the front view condition in the body task and the turned condition in the letter task were expected to lead to comparably increased response times and/or error rates as compared, respectively, to the back-viewing bodies and unturned letters [55], reflecting the need for the supplementary mental transformation process.

Body and letter stimuli, which had the same dimensions along the vertical and the horizontal axes (600 × 600 pixels), were presented at the center of the screen until a response was given. A 1 sec interval was allowed between trials. Participants had to respond to each trial, as fast and accurately as possible, by pressing the right or the left button of the mouse, which corresponded, respectively, to a right or left judgement response. The body and letter stimuli were presented in separate 64-trial blocks, with a between-subject counterbalance in block order. Moreover, in each block, 32 trials requiring a mental transformation (i.e., front-viewing bodies or turned letters) and 32 trials not requiring it (back-viewing bodies; unturned letters) were randomly presented. In a similar vein, the two genders of the manikin or the two types of letter F as well as left and right response trials were randomly presented within each block. The body and letter transformation judgements were matched in terms of complexity and axis of mental transformation [55].

### 2.4. Visual Body Recognition Task

The visual body recognition task required participants to perform delayed same-different judgments on color pictures of body postures. Stimuli were created by photographing two boys and two girls aged 8 years while showing six different body postures with various displacements of lower and upper limbs. The postures had no emotional content or symbolic meaning. All children were wearing the same grey/blue or pink/yellow t-shirts and shorts and were photographed while displaying the same set of body postures. The pictures were taken from a frontal or sideway perspective, displayed on a white background, and subtended a 539 × 737-pixel area. Each of the 24 original pictures was digitally edited using the Adobe Photoshop software (Adobe Systems Inc.), in order to create a paired stimulus by combining the upper half of the body with the lower half of the body picture of a different model, matched for gender and posture. We obtained 24 pairs, with the two stimuli in each pair having the same upper half but a different lower half. Participants were presented with a sequence of two body stimuli and were required to detect whether the upper part of the second stimulus was the same or different as compared to the upper part of the first stimulus. In the same-response trials, each stimulus was presented with the matching stimulus of the pair that had the same upper half but a different lower half. Conversely, in the different-response trials, each stimulus was presented with a stimulus of a different pair created from the same models and having different upper and lower parts. According to the previous literature [79], we adopted a partial design with a 2 : 1 proportion of same- vs. different-response trials, resulting in 24 same-response trials and 12 different-response trials (but see Richler and Gauthier [101]). With the aim of evaluating the inversion and the composite illusion effects, stimuli were presented inverted or upright (*orientation condition*) and aligned or misaligned (*alignment condition*). Considering 24 same- and 12 different-response trials per condition, a total of 144 trials was administered. For the orientation manipulation, inverted stimuli were rotated at 180° along the horizontal axis (i.e., reversed upside down). For the alignment manipulation, misaligned stimuli were obtained by shifting along the horizontal axis the lower body part to the right starting at the middle of the upper body half. To avoid discontinuities in body figures, no gap was left between lower and upper body parts, in line with a previous study documenting reliable composite illusion effect for bodies [67]. Since previous studies have reported reduced [62] or absent inversion effects for headless bodies [102, 103], it was decided to maintain children's face but to scramble it in order to make impossible face identity discrimination [61].

The experiment was divided into 6 different blocks, each one consisting of 16 same- and 8 different-response trials. Before starting the experiment, participants received both oral and written instructions on the task. In order to verify comprehension of task rules and methods, they were presented with 8 practice trials, which were then excluded from statistical analyses. Each trial started with the presentation of a central fixation cross lasting 1.000 ms. Then, the first stimulus was presented for 1.500 ms, followed by a random-dot mask (7.6° × 7.6° in size; duration between 550 and 690 ms) created by scrambling body stimuli. The probe stimulus appeared immediately after the disappearance of the mask and remained on the screen or until a response was given or for a maximum of 3.500 ms. Participants had a maximum interval of 5.000 ms from the onset of the second stimulus to respond. In each trial, the paired stimuli had the same orientation and alignment but had different lower parts, while the upper parts could be either the same or different. Participants had to respond as quickly and accurate as possible by pressing the left or the right key on the computer mouse, corresponding, respectively, to a same or a different response. The following trial appeared after an intertrial interval of 2.500 ms.

### 2.5. NEPSY-II

The NEPSY-II is a comprehensive neuropsychological battery designed to evaluate six different cognitive domains in children aged 3-16 years [99]. In our study, we administered the two subtests belonging to the social perception domain. The first is the theory of mind (ToM) subtest, which is composed of two parts resulting in one raw score. The verbal part assesses the ability to understand mental constructs, such as beliefs and intentions, and how other people could have thoughts, emotions, and perspectives different from ours. The contextual part evaluates the ability to infer others' emotion by social context. The second subtest is named affect recognition and assesses the ability to recognize affective states from different photos of children's faces. Raw scores were transformed into *z* scores according to the distribution of the age-matched normative values for the Italian sample [100] and then converted into scaled scores (mean 10; SD 3). This procedure avoided the approximation at the low or high extremes which is used in standard normative conversion tables, thus including also negative numbers for performance lower than -3.33 SD from the mean.

### 2.6. Data Reduction and Statistical Analysis

The individual mean percentages of correct responses (Accuracy) and reaction times (RTs) of correct responses for the different conditions of the two tasks were inserted into statistical analyses. For both tasks, trials with anticipated or out-of-time responses (RT < 150 or >0 ms) were not considered. Moreover, for the *visual body recognition task*, only the same-condition trials were considered, as suggested in previous studies on the composite illusion effect [65, 79]. In order to control for possible speed-accuracy tradeoff effects, an Inverse Efficiency (IE) index was calculated as the ratio between RTs and Accuracy for each condition of the two tasks. The IEs were entered in a 3-way mixed-model, repeated-measures 2 × 2 × 2 ANOVA for each task, using Group as a between-subject factor. The within-subject variables were Stimuli (body *vs.* letter) and Transformation (required *vs.* nonrequired), for the *visuospatial imagery task*, or Alignment (aligned *vs.* nonaligned) and Orientation (upright *vs.* inverted), for the *visual body recognition task*. Multiple-way interactions were analyzed with Tukey's post hoc test correction for multiple comparisons. With the aim to explore the within-subject effects, planned 2-way ANOVAs were conducted in each group for the two tasks. In order to verify the effects of demographic and clinical variables on the execution of the two tasks, we ran regression analyses within the SpD group. In particular, gender, age, functional asymmetry, presence of CVI, FSIQ, scores at GMFCS and MACS, and mean score at NEPSY-II social perception subtests were inserted as predictors in regression models. Dependent variables were three delta measures considered as indexes of the first-person and object-based transformations and of the composite illusion effect. Indeed, for the *visuospatial imagery task*, we calculated for the body and for the letter tasks the delta in IE between the congruent condition and the one requiring the mental transformation. For the *visual body recognition task*, we first computed the difference between the IE for the aligned *vs.* nonaligned conditions when stimuli were presented upright or inverted and then subtracted the IE difference for the inverted stimuli from that for the upright stimuli. The resulting delta reflected how strong was the impact of alignment for the upright as compared to the inverted stimuli, thus allowing us to control for the impact of the effect of the interaction between Alignment and Orientation. Finally, to understand whether children and adolescents with SpD were impaired in social perception abilities, we used one-sample *t*-test (two-tailed) to compare their scaled scores on NEPSY-II scales with the normative values (*M* = 10). For all analyses, the significance threshold was set at *p* < 0.05. Effect sizes were reported as partial eta squared (*η*_*p*_^2^), using conventional cutoffs for *η*_*p*_^2^ = 0.01, 0.06, and 0.14 for small, medium, and large effect sizes, respectively [104]. Moreover, data were reported as mean and standard error of the mean (SEM).

## 3. Results

### 3.1. Visuospatial Imagery Task

Raw data for Accuracy and RT are reported in Table 3.

**Figure 2:**
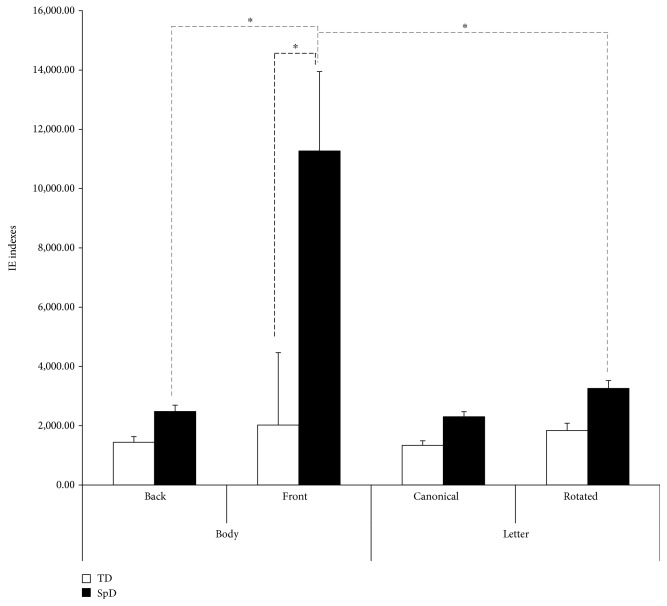
Inverse Efficiency (IE) indexes for each condition of the *visuospatial imagery task* for the two groups. Bars indicate standard error of the mean of measurements in 64 bodies and 64 letter trials for 25 SpD (spastic diplegia) and 30 TD (typical development) participants; thinner dotted grey lines indicate within-group comparisons, and thicker dotted black line indicates between-group comparison. Asterisks indicate significant *p* < 0.05.

Five SpD patients were not able to perform the task, as revealed by a below-chance performance in the congruent condition, which did not require any mental transformation; thus, they were excluded from further analyses. The 3-way 2 Stimuli × 2 Transformation × 2 Group ANOVA conducted on the IE indexes revealed a significant between-subject Group effect (*F*_1,53_ = 11.771, *p* = 0.001, *η*_*p*_^2^ = 0.182) with SpD patients overall less able in performing the task. Moreover, the main effects of Stimulus and Transformation and their respective 2-way interactions with Group were significant (all *F*_1,53_ > 4.663; all *p* < 0.035) and were better qualified by a significant 3-way interaction Stimuli × Transformation × Group (*F*_1,53_ = 4.475, *p* = 0.039, *η*_*p*_^2^ = 0.078). Tukey post hoc tests revealed that the first-person mental transformation performance of SpD patients was lower than all other cells of the design (all *p* < 0.003). Indeed, when body stimuli required a mental transformation, SpD patients presented an impairment (*p* < 0.001; 11,267.770 ± 2,676.334) compared to healthy children (2,022.730 ± 2,443.148). No significant differences emerged for the object-based mental transformation of the rotated letter (*p* = 0.994), with a comparable IE index in the SpD (3,258.480 ± 266.913) and the TD (1,840.890 ± 243.657) groups. Notably, for SpD children, the deficit in operating the mental transformation was specific for body stimuli, since their performance for the front-viewing bodies was worse than that for turned letters (*p* = 0.003), while no differences between the two transformed stimuli were found in the TD group (*p* > 0.99). The planned, follow-up 2-way ANOVA within the control group confirmed the effect of Transformation (*F*_1,29_ = 31.559, *p* < 0.001, *η*_*p*_^2^ = 0.521), reflecting the task difficulty when a mental rotation is required, but the main effect of Stimulus and the interaction between Stimulus and Transformation were nonsignificant (all *F*_1,29_ < 0.814; all *p* > 0.374). The Transformation effect was similarly found in SpD patients (*F*_1,24_ = 5.897, *p* = 0.023, *η*_*p*_^2^ = 0.197), but the Stimulus effect was also significant (*F*_1,24_ = 4.280, *p* = 0.049, *η*_*p*_^2^ = 0.151), indicating a greater impairment in performing the mental rotation with body stimuli (6,875.716 ± 1,980.053) rather than with letters (2,780.714 ± 217.802). However, the interaction between Stimulus and Transformation was nonsignificant (*F*_1,24_ = 3.803, *p* = 0.063, *η*_*p*_^2^ = 0.134). Globally, these results suggested that pediatric patients with SpD were more impaired in motor but not in visual imagery as compared to TD children. IE indexes for each condition of the task for the two groups are reported in Figure 2.

The regression analyses within the SpD group did not reveal any significant models for both the body and the letter transformations. Thus, demographic and clinical features could not explain the specific deficit shown by the SpD group in operating the mental rotation with body stimuli, although it is of note that gender was found to be a significant predictor for the performance in the first-person transformation task, with better performance for girls. The results of the regression models are reported in Table 4.

### 3.2. Visual Body Recognition Task

Raw data for Accuracy and RTs for the visual body recognition task are reported in Table 5.

**Figure 3:**
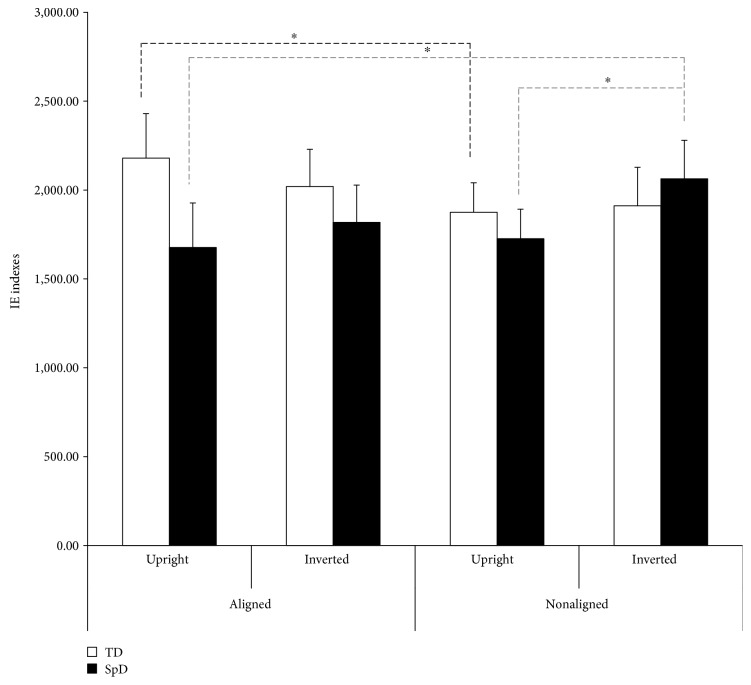
Inverse Efficiency (IE) indexes for each condition of the *visual body recognition task* for the two groups. Bars indicate standard error of the mean of measurements of 16 same-response trials in six blocks (*n* = 96) for 30 SpD and 30 TD participants; dotted black line indicates within-TD group comparisons, and dotted grey lines indicate within-SpD group comparisons. Asterisks indicate significant *p* < 0.05.

The analyses of the IE indexes did not reveal a significant effect of Group (*p* = 0.532) but a significant 2-way interactionAlignment × Orientation (*F*_1,58_ = 4.592, *p* = 0.036, *η*_*p*_^2^ = 0.073). These findings suggested that both inversion and composite illusion effects were present in the whole sample. However, the 2-way interactions Alignment × Group (*F*_1,58_ = 4.373, *p* = 0.041, *η*_*p*_^2^ = 0.070) and Orientation × Group (*F*_1,58_ = 6.800, *p* = 0.012, *η*_*p*_^2^ = 0.105) were significant, advising that the use of configural and holistic processing was different in the two groups. The planned follow-up ANOVA confirmed that SpD patients and TD children performed the task differently. Indeed, in the control group, a significant 2-way interaction Alignment × Orientation (*F*_1,29_ = 7.542, *p* = 0.010, *η*_*p*_^2^ = 0.206) was found, in keeping with the presence of holistic and configural processing in body perception. Tukey post hoc tests revealed that the TD group had a better performance (*p* < 0.001) with the upright nonaligned stimuli (1,874.841 ± 181.148) than with the upright aligned ones (2,180.161 ± 308.335). Notably, this was not found for inverted stimuli, since the inversion of the stimuli disrupts the first-level configural processing and, consequently, any higher-order holistic perception, resulting into a nonsignificant alignment effect for inverted stimuli. In the SpD group, a significant effect of Orientation was found (*F*_1,29_ = 6.386, *p* = 0.017, *η*_*p*_^2^ = 0.180), with the IE being lower for upright than inverted stimuli, thus pointing to spared inversion effect for (and first-order configural processing of) body stimuli. However, the 2-way interaction Alignment × Orientation was not significant (*p* = 0.146), showing that no evidence emerged for the composite illusion effect and that the use of holistic processing for body stimuli is altered in SpD children. IE indexes for each condition of the task for the SpD and the TD group are depicted in Figure 3.

Notably, the regression model with demographic and clinical data as predictors and the delta estimate of the Alignment-Inversion interaction effect as a dependent variable was not significant and no predictor showed a significant association with the dependent variable. This excludes that the absence of composite illusion effect in SpD patients could be attributed to their general demographic and clinical variables. Coefficients and *p* values of the regression model are reported in Table 6.

### 3.3. NEPSY-II

One-sample *t*-tests revealed that the SpD group obtained scaled scores significantly lower than the normative mean in both the ToM (*t*_25_ = −3.671; *p* = 0.001) and the affect recognition (*t*_25_ = −6.204; *p* < 0.001) subtests.

## 4. Discussions

In this study, we explored whether children and adolescents with SpD could present alterations at the mental transformation and perceptual levels of body representation. In particular, we compared their performance with that of an age-matched control sample in two different tasks with the aim to evaluate motor and visual imagery abilities and perceptual processing strategies for body stimuli. In the *visuospatial imagery task*, we asked participants to give left-right judgments relative to whole body or letter stimuli presented in rotated or canonical positions, thus activating or not the motor or visual imagery strategies. To explore alterations at a perceptual level of body representation, participants were administered a *visual body recognition* task designed to evaluate the presence of the inversion and the composite illusion effects for others' body, which in turn are considered to be measures of configural and holistic processing. Finally, social perception abilities were assessed in the SpD group using the ToM and the affect recognition subtests of the NEPSY-II. Results revealed that, overall, children with SpD had more difficulties than the control sample in the mental imagery tasks either bodies or objects. Nevertheless, a specific deficit in operating a first-person mental transformation emerged for the SpD group. In the body perception task, data pointed to the presence of the low-level configural processing in both groups, while no evidence for the use of holistic processing was found in the SpD patients. Despite no clear association between social abilities and the performance in the tasks emerged, NEPSY-II assessment revealed that SpD children had lower scores than normative values in both the social perception subtests and particularly in the affect recognition one.

Importantly, the performance of the SpD group in the tasks was not explained by demographic and clinical variables, with the notable exception of gender. Thus, our findings of altered motor imagery and of nonuse of holistic processing of body stimuli were not related to limited intellectual abilities or to basic visual and motor impairments, but they may reflect specific deficits in high-level motor cognition, nor can the effects be ascribed to the interaction between limited cognitive abilities in SpD and task difficulty. Indeed, patients failed more the first-person mental transformation tasks than the third-person mental transformation one, albeit the two tasks are comparably challenging in the TD individuals tested in this study as well as in previous studies [55, 98]. In a similar vein, inversion and composite illusion exert comparable effects on body recognition performance. Still we found that SpD patients presented with spared body inversion effects but altered composite illusion effects. In sum, the task selectivity of patients' impairments makes it unlikely that general cognitive impairments or general clinical conditions may explain the body-specific deficits.

### 4.1. Visuospatial Imagery Task

While hemiplegia imagery is usually assessed using body part (i.e., hand) stimuli [40, 105], here, we used whole bodies, in line with the bilateral motor deficits showed by SpD patients. Nevertheless, the results are in keeping with the findings from patients with hemiplegia, suggesting an impairment in using motor imagery [48, 106]. However, in SpD children, a visual imagery deficit was also found, while this ability seems to be spared in hemiplegia [36]. This general difficulty in executing the task, independently from the stimulus and imagery strategy, could be connected to the core visuospatial deficits shown by patients with SpD [7, 8]. Indeed, an impairment in visual mental imagery was documented in adolescents with SpD and it was associated with perceptual deficits [87]. It should be noted that visual imagery and motor imagery are different cognitive processes [36, 44] even if they may share common aspects [107]. In particular, a general visuospatial process may be involved in both tasks [56], but visual imagery is strictly connected to visuoperceptual abilities [108], while motor imagery involves the simulation of a movement without a motor output and is related to motor experience and motor planning [36, 109]. Thus, while the general difficulties of SpD patients in responding to both body and letter stimuli may reflect their visuospatial processing abilities [7, 8], a specific impairment in operating the mental rotation of the whole body was found. This result confirmed the role of one's own embodied motor experience for motor imagery, especially when a first-person mental transformation is required [110]. Accordingly, Corti and colleagues found that pediatric patients with brain tumors performed overall worse than children with TD at the *visuospatial imagery task* but only patients with damages to areas involved in motor simulation processes (i.e., cerebellum) showed a selective alteration in using the first-person mental transformation for body stimuli [98]. Indeed, this mental process involves own body schema, while this is not observed for object rotation [111]. Thus, since patients with hemiplegia have difficulties in the motor imagery of the affected limb [50, 51], the biased motor experience of both body sides in SpD patients may alter their general body schema, resulting in specific deficits in performing first-person whole-body mental transformations.

### 4.2. Visual Body Recognition Task

Visual perception of human bodies relies on specific cognitive and neural mechanisms that are different from those activated by objects [58, 62]. In particular, face and body stimuli are processed using configural and holistic processing, as revealed, respectively, by the inversion [59, 112] and the composite illusion [78, 79] effects. Although the presence of composite illusion for body stimuli is still controversial [65–67, 76, 113], our results support the reliability of both effects in body perception. Notably, we found these effects in a pediatric population, suggesting an early refinement of perceptual processes for bodies [114] as it has been found in infancy for faces [115–117].

Furthermore, it has been demonstrated that visual and embodied expertise, connected to motor and proprioceptive information, could have independent effects on the development of configural processing for body perception [88]. Since SpD children could be impaired in both visuospatial abilities and motor experience, our hypothesis was that a deficit in configural and holistic processing should be found in this sample. Partially in contrast with this view, both TD and SpD groups showed a reliable inversion effect for body stimuli, suggesting that configural processing could be spared in SpD children. Conversely, the SpD sample did not present a reliable composite illusion effect, pointing to a selective deficit in holistic processing. This result is in line with research on pediatric patients with brain tumors [98] and suggests that a dissociation between configural and holistic processing could be presented for body as documented for faces [68]. While these perceptual processes could mostly rely on similar mechanisms [112], it has been suggested that holistic processing, as measured by the composite illusion, may be seen as a failure of selective attention [118]. Indeed, at least for faces, composite illusion could rely on an attention-dependent mechanism that can integrate spatially separated face parts [119–121]. The attentional deficits shown by SpD children [11] may interfere with this mechanism, resulting in alterations of holistic processing of body stimuli. Though we controlled that the results were not imputable to general cognitive abilities, we did not adopt a specific measure for attention and executive functions. Thus, further research is requested to verify whether attentional deficits could alter the use of holistic processing of body stimuli.

Importantly, however, configural and holistic processes for body stimuli seem to involve not only occipitotemporal visual regions [73, 77, 122] but also frontal motor areas [123]. Indeed, alterations in motor activity resulting from transcranial magnetic stimulation affected the processing of upright, but not inverted, whole-body stimuli, disrupting the body inversion effect in healthy individuals [124]. In a similar vein, early lesions to motor areas, associated with limitations in motor experience, could impact on the development of selective perceptual processes for whole bodies in SpD children. Since holistic processing is associated with a refined perception of others' bodies and the bodies of other individuals convey important information on their behavioral intentions and feelings, an altered holistic processing in SpD may lead to more widespread difficulties in social relationships.

### 4.3. Social Perception Abilities

A deficit in social perception, assessed through the NEPSY-II battery, was found in SpD children and adolescents. As concerns the ToM subtest, patients obtained lower scores than normative values. Other works documented that CP patients could present impairments in specific aspects of ToM, particularly in false-beliefs tasks [125]. While these deficits have been mostly linked to alterations in executive functions [126], other aspects, such as language abilities, motor diseases, and life experience, have to be considered [127]. Indeed, SpD children could present impairments in executive functions [12, 13], but they also have a biased motor experience [4], which could impact on the development of ToM abilities. Indeed, an even greater deficit of SpD patients was found in the affect recognition subtest, which assesses emotion recognition abilities. While evidence of emotion processing difficulties in SpD children and adolescents is still sparse [11], these findings may suggest a link between motor impairments, embodied experience, and social perception [128–130]. Body is a crucial medium not just for emotion expressions but also to understand others' internal states [131]. Accordingly, the first-person mental transformation that required aligning one's own body with other's perspective has been suggested to be the first step of higher-level understanding of others' intention, beliefs, and affects [132]. In line with this data, adolescents with autism were found to be impaired in using their own bodily information to take others' perspective [133]. In a similar vein, experienced schizotypal body schema alterations were associated to a dysfunction in mental own-body transformation [54]. Thus, limitations in using one's own body to align with others' view and in others' body perception could impact on social cognition abilities [132]. However, it is noteworthy that we did not find a direct correlation between social perception abilities and the performance in the tasks of the SpD group. Thus, our hypothesis of a strict link between motor impairments, altered motor representations, and deficits in social perception should be verified in future research.

### 4.4. Limitations

The conclusions that can be drawn from this work need to be considered in the light of the limitations. First, a great prevalence of males was recruited in the SpD group. While this data is in accordance with the previous literature [95], we could not match the two samples for gender. To verify the effect of the gender on the results in the TD group, we conducted a series of *t*-test with IE indexes in the two tasks as dependent variables. In keeping with a previous study [55], no significant differences emerged (all *t*_28_ < 1.280 and >-0.837, all *p* > 0.211), suggesting that there was no modulation of gender on task performance. Although no regression model was found to be significant within the clinical group, a gender effect was reliable in operating the mental rotation of the body, suggesting that girls were less affected than boys in operating a transformation. This result could reflect a sampling bias, with only a limited number of girls in our sample; thus, our conclusions on the motor imagery abilities in patients with SpD should be limited to boys. About the *visuospatial imagery task*, the 180° rotated perspective condition (i.e., the front view) has been recently issued in its ability to activate own-body mental rotation [134]. Indeed, for the 180° rotations, children could answer inverting the left-right and front-back axis without rotating their mental position. However, it should be noted that this kind of stimulus presentation has shown its reliability in studies on pediatric populations [55], specifically in dissociating object- and viewer-transformation ability [98, 135]. As regards the *visual body recognition task*, both inversion [70] and composite illusion effects [118] have been criticized for their ability to selectively interfere with configural and holistic processing. Moreover, it has been argued that a complete design rather than a partial one should be preferred for the composite illusion task [101, 136], although researches on holistic processing in pediatric samples have usually adopted the latter method [137]. As concerns the NEPSY-II assessment, it should be noted that the mean score obtained by our SpD sample in the ToM subtest is at the inferior limit of the normative range [100]. Thus, our results do not clearly support the view of a generalized ToM deficit in SpD children and the hypothesis of a link between motor impairments and social deficit in SpD pediatric population should be limited to affect recognition. Finally, we did not control for the extent of brain lesions in our SpD sample; therefore, we could not directly verify our hypotheses on associations between specific cerebral damage and deficits in body representation.

## 5. Conclusions

The findings of the present study indicated that children and adolescents with SpD could present impairments at the levels of body schema and of body perceptual processing. In particular, while a general deficit in using visual imagery was found in line with visuospatial deficits showed by these patients, the results pointed to a specific impairment in performing first-person whole-body mental transformations (i.e., motor imagery). Moreover, pediatric patients with SpD exhibited difficulties in body-related perceptual processing. Indeed, an alteration in using holistic processing but not configural processing for body stimuli was found. Finally, NEPSY-II assessment revealed that our SpD group presented social perception deficits, particularly in affect recognition. Despite literature is still scant, our results suggested that a possible association between motor impairments, body representation deficits, and social difficulties has to be taken into account not only in future research but also in the evaluation and treatment for this clinical sample. New methodologies of educative and rehabilitative interventions focused on the empowerment of an adequate perception and representation of bodies should be proposed and tested to facilitate the embodiment of sensorial and motor states of other people and, thus, to improve social perception competencies of children and adolescents with SpD.

## Figures and Tables

**Table 1:** Clinical information for the SpD group according to Rosenbaum et al. [5].

(1) Motor abnormalities	
(a) Nature and typology of the motor disorders	Spasticity
(b) Functional motor abilities	
MACS	
Mean (SD)	1.50 (0.63)
Range	1-3
GMFCS	
Mean (SD)	2.40 (0.97)
Range	1-4
(2) Accompanying impairments	
Presence of epilepsy (*N*)	3
FSIQ	
Mean (SD)	79.60 (19.81)
Range	41-115
Presence of hearing impairments (*N*)	0
Presence of Cortical Visual Impairment (*N*)	6
(3) Anatomic distribution and neuroimaging findings	
(a) Anatomic distribution	
Bilateral (*N*)	30
With right asymmetry (*N*)	6
With left asymmetry (*N*)	8
(b) Neuroimaging findings	
Periventricular leukomalacia (*N*)	9
Not available (*N*)	21
(4) Cause and timing	
Periventricular leukomalacia in preterm birth (*N*)	8
Periventricular leukomalacia in at-term birth (*N*)	1
Hydrocephalus (*N*)	1
Unclear (*N*)	20

SD = standard deviation; MACS = Manual Ability Classification System; GMFCS = Gross Motor Function Classification System; FSIQ = full-scale intelligent quotient.

**Table 2:** Demographic information and scores at the social perception subtests of NEPSY-II.

	SpD group	TD group
*Demographic information*		
*N*	30	30
Male : female	26 : 4	14 : 16
Age (years)		
Mean (SD)	11.65 (3.02)	11.47 (3.29)
Range	7.37-18.00	8.00-18.00
*Social perception*		
Theory of mind (scaled scores)		
Mean (SD)	7.15 (3.86)	
Range	1-14	
Affect recognition (scaled scores)		
Mean (SD)	5.93 (3.46)	
Range	1-15	

SD = standard deviation; SpD = spastic diplegia; TD = typical development.

**Table 3:** Accuracy and reaction time in each condition of the *visuospatial imagery task* for the two groups. Data are reported as mean ± SEM.

Stimulus	Transformation	Accuracy (%)		RTs (ms)	
		TD children	SpD patients	TD children	SpD patients
Body	Nonrequired	90.77 ± 1.65	77.00 ± 4.01	1,280.16 ± 80.68	1,696.28 ± 90.53
	Required	88.20 ± 1.73	51.04 ± 7.21	1,720.66 ± 135.77	1,819.65 ± 140.39

Letter	Nonrequired	93.73 ± 1.63	70.36 ± 3.30	1,226.44 ± 89.71	1,556.93 ± 126.53
	Required	87.33 ± 2.81	50.08 ± 1.16	1,473.59 ± 96.11	1,599.06 ± 136.33

**Table 4:** Results of the regression models within the SpD group for the body and the letter transformations.

	Dependent variables					
	Body transformation delta			Letter transformation delta		
	*N* = 25			*N* = 25		
	Adj *R*^2^	F8, 16	*p* level	Adj *R*^2^	F8, 16	*p* level
	0.313	2.364	0.068	-0.337	0.240	0.975
*Independent variables*	*B*	*t*	*plevel*	*B*	*t*	*plevel*
Gender	-0.425	-2.223	0.041	-0.093	0.350	0.731
Age	0.283	1.437	0.170	0.057	0.208	0.838
Functional asymmetry	0.040	0.200	0.844	-0.352	-1.268	0.223
CVI	-0.113	-0.532	0.602	0.059	0.200	0.844
FSIQ	-0.471	-2.078	0.054	0.229	0.724	0.480
GMFCS	-0.008	-0.034	0.973	-0.077	-0.243	0.811
MACS	-0.049	-0.228	0.823	-0.008	-0.026	0.980
Mean social perception	0.029	0.149	0.883	-0.033	-0.119	0.907

CVI = Cortical Visual Impairment; FSIQ = full-scale intelligent quotient; GMFCS = Gross Motor Function Classification System; MACS = Manual Ability Classification System.

**Table 5:** Accuracy and reaction time in each experimental condition for the two groups. Data are reported as mean ± SEM.

Stimulus		Accuracy (%)		RTs (ms)	
Alignment	Orientation	TD children	SpD patients	TD children	SpD patients
Aligned	Canonical	79.53 ± 3.14	82.33 ± 3.55	1,497.34 ± 100.62	1,261.36 ± 78.92
	Inverted	81.13 ± 3.10	80.68 ± 3.14	1,474.97 ± 94.53	1,336.45 ± 75.81

Nonaligned	Canonical	81.90 ± 2.76	82.60 ± 3.33	1,426.66 ± 92.54	1,308.17 ± 67.88
	Inverted	82.97 ± 2.45	77.43 ± 4.36	1,502.38 ± 94.82	1,315.70 ± 86.14

**Table 6:** Results of the regression model within the SpD group for the Alignment-Inversion interaction effect.

	Dependent variable		
	Alignment-Inversion interaction effect		
	*N* = 30		
	Adj *R*^2^	F8, 21	*p* level
	-0.089	0.704	0.685
*Independent variables*	*B*	*t*	*p* level
Gender	-0.238	-1.122	0.274
Age	-0.048	-0.204	0.840
Functional asymmetry	-0.299	-1.327	0.199
CVI	-0.186	-0.751	0.461
FSIQ	-0.062	-0.243	0.810
GMFCS	-0.188	-0.726	0.475
MACS	0.137	0.524	0.606
Mean social perception	-0.128	-0.566	0.577

CVI = Cortical Visual Impairment; FSIQ = full-scale intelligent quotient; GMFCS = Gross Motor Function Classification System; MACS = Manual Ability Classification System.

## Data Availability

Data are collected in a protected database and are anonymized, as a research member assigned to each participant an identity number that substitutes the name. Participants' parents give written informed consent to anonymized data use. The anonymized data are available at request by sending an email to Dr. Cosimo Urgesi (cosimo.urgesi@uniud.it).

## References

[1] Oskoui M., Coutinho F., Dykeman J., Jetté N., Pringsheim T. (2013). An update on the prevalence of cerebral palsy: a systematic review and meta-analysis. *Developmental Medicine and Child Neurology*.

[2] Fennell E. B., Dikel T. N. (2001). Cognitive and neuropsychological functioning in children with cerebral palsy. *Journal of Child Neurology*.

[3] Odding E., Roebroeck M. E., Stam H. J. (2006). The epidemiology of cerebral palsy: incidence, impairments and risk factors. *Disability and Rehabilitation*.

[4] Straub K., Obrzut J. E. (2009). Effects of cerebral palsy on neuropsychological function. *Journal of Developmental and Physical Disabilities*.

[5] Rosenbaum P., Paneth N., Leviton A. (2007). A report: the definition and classification of cerebral palsy April 2006. *Developmental Medicine and Child Neurology*.

[6] Fazzi E., Bova S. M., Uggetti C. (2004). Visual-perceptual impairment in children with periventricular leukomalacia. *Brain & Development*.

[7] Pagliano E., Fedrizzi E., Erbetta A. (2007). Cognitive profiles and visuoperceptual abilities in preterm and term spastic diplegic children with periventricular leukomalacia. *Journal of Child Neurology*.

[8] Ego A., Lidzba K., Brovedani P. (2015). Visual-perceptual impairment in children with cerebral palsy: a systematic review. *Developmental Medicine and Child Neurology*.

[9] Eliasson A.-C., Krumlinde-Sundholm L., Rösblad B. (2006). The Manual Ability Classification System (MACS) for children with cerebral palsy: scale development and evidence of validity and reliability. *Developmental Medicine and Child Neurology*.

[10] Beckung E., Hagberg G. (2002). Neuroimpairments, activity limitations, and participation restrictions in children with cerebral palsy. *Developmental Medicine and Child Neurology*.

[11] Di Lieto M. C., Brovedani P., Pecini C. (2017). Spastic diplegia in preterm-born children: executive function impairment and neuroanatomical correlates. *Research in Developmental Disabilities*.

[12] Korkman M., Mikkola K., Ritari N. (2008). Neurocognitive test profiles of extremely low birth weight five-year-old children differ according to neuromotor status. *Developmental Neuropsychology*.

[13] Pirila S., van der Meere J., Korhonen P. (2004). A retrospective neurocognitive study in children with spastic diplegia. *Developmental Neuropsychology*.

[14] Imms C. (2008). Children with cerebral palsy participate: a review of the literature. *Disability and Rehabilitation*.

[15] Pavlova M., Sokolov A. N., Birbaumer N., Krägeloh-Mann I. (2008). Perception and understanding of others’ actions and brain connectivity. *Journal of Cognitive Neuroscience*.

[16] Cioni G. (2000). Correlation between visual function, neurodevelopmental outcome, and magnetic resonance imaging findings in infants with periventricular leucomalacia. *ADC Fetal & Neonatal*.

[17] Guzzetta A., Tinelli F., Bancale A., Cioni G. (2010). Visual and oculomotor disorders. *The Spastic Forms of Cerebral Palsy*.

[18] Pavlova M., Sokolov A., Krägeloh-Mann I. (2007). Visual navigation in adolescents with early periventricular lesions: knowing where, but not getting there. *Cerebral Cortex*.

[19] Pavlova M. A., Krägeloh-Mann I. (2013). Limitations on the developing preterm brain: impact of periventricular white matter lesions on brain connectivity and cognition. *Brain*.

[20] Hirai M., Hiraki K. (2005). An event-related potentials study of biological motion perception in human infants. *Cognitive Brain Research*.

[21] Pavlova M. A. (2012). Biological motion processing as a hallmark of social cognition. *Cerebral Cortex*.

[22] Morin A. (2006). Levels of consciousness and self-awareness: a comparison and integration of various neurocognitive views. *Consciousness and Cognition*.

[23] Porciello G., Bufalari I., Minio-Paluello I., Di Pace E., Aglioti S. M. (2018). The ‘enfacement’ illusion: a window on the plasticity of the self. *Cortex*.

[24] Berlucchi G., Aglioti S. M. (2010). The body in the brain revisited. *Experimental Brain Research*.

[25] Avenanti A., Candidi M., Urgesi C. (2013). Vicarious motor activation during action perception: beyond correlational evidence. *Frontiers in Human Neuroscience*.

[26] Urgesi C., Candidi M., Avenanti A. (2014). Neuroanatomical substrates of action perception and understanding: an anatomic likelihood estimation meta-analysis of lesion-symptom mapping studies in brain injured patients. *Frontiers in Human Neuroscience*.

[27] Rizzolatti G., Craighero L. (2004). The mirror-neuron system. *Annual Review of Neuroscience*.

[28] Barsalou L. W. (2008). Grounded cognition. *Annual Review of Psychology*.

[29] Gallese V., Sinigaglia C. (2011). What is so special about embodied simulation?. *Trends in Cognitive Sciences*.

[30] Rizzolatti G., Sinigaglia C. (2010). The functional role of the parieto-frontal mirror circuit: interpretations and misinterpretations. *Nature Reviews Neuroscience*.

[31] Bonner M. J., Hardy K. K., Willard V. W., Anthony K. K., Hood M., Gururangan S. (2008). Social functioning and facial expression recognition in survivors of pediatric brain tumors. *Journal of Pediatric Psychology*.

[32] de Gelder B. (2009). Why bodies? Twelve reasons for including bodily expressions in affective neuroscience. *Philosophical Transactions of the Royal Society B: Biological Sciences*.

[33] Soria Bauser D., Thoma P., Aizenberg V., Brüne M., Juckel G., Daum I. (2012). Face and body perception in schizophrenia: a configural processing deficit?. *Psychiatry Research*.

[34] Wann J. P. (1991). The integrity of visual-proprioceptive mapping in cerebral palsy. *Neuropsychologia*.

[35] Chinier E., N’Guyen S., Lignon G., Ter Minassian A., Richard I., Dinomais M. (2014). Effect of motor imagery in children with unilateral cerebral palsy: fMRI study. *PLoS One*.

[36] Crajé C., van Elk M., Beeren M., van Schie H. T., Bekkering H., Steenbergen B. (2010). Compromised motor planning and motor imagery in right hemiparetic cerebral palsy. *Research in Developmental Disabilities*.

[37] Jongsma M. L. A., Baas C. M., Sangen A. F. M. (2016). Children with unilateral cerebral palsy show diminished implicit motor imagery with the affected hand. *Developmental Medicine and Child Neurology*.

[38] Mutsaarts M., Steenbergen B., Bekkering H. (2007). Impaired motor imagery in right hemiparetic cerebral palsy. *Neuropsychologia*.

[39] Frassinetti F., Fiori S., D’Angelo V. (2012). Body knowledge in brain-damaged children: a double-dissociation in self and other’s body processing. *Neuropsychologia*.

[40] Frassinetti F., Maini M., Benassi M., Avanzi S., Cantagallo A., Farnè A. (2010). Selective impairment of self body-parts processing in right brain-damaged patients. *Cortex*.

[41] Munzert J., Lorey B., Zentgraf K. (2009). Cognitive motor processes: the role of motor imagery in the study of motor representations. *Brain Research Reviews*.

[42] Mulder T. (2007). Motor imagery and action observation: cognitive tools for rehabilitation. *Journal of Neural Transmission*.

[43] Cabral-Sequeira A. S., Coelho D. B., Teixeira L. A. (2016). Motor imagery training promotes motor learning in adolescents with cerebral palsy: comparison between left and right hemiparesis. *Experimental Brain Research*.

[44] Madan C. R., Singhal A. (2012). Motor imagery and higher-level cognition: four hurdles before research can sprint forward. *Cognitive Processing*.

[45] Miller L. E., Saygin A. P. (2013). Individual differences in the perception of biological motion: links to social cognition and motor imagery. *Cognition*.

[46] Steenbergen B., Jongbloed-Pereboom M., Spruijt S., Gordon A. M. (2013). Impaired motor planning and motor imagery in children with unilateral spastic cerebral palsy: challenges for the future of pediatric rehabilitation. *Developmental Medicine and Child Neurology*.

[47] Williams J., Anderson V., Reddihough D. S., Reid S. M., Vijayakumar N., Wilson P. H. (2011). A comparison of motor imagery performance in children with spastic hemiplegia and developmental coordination disorder. *Journal of Clinical and Experimental Neuropsychology*.

[48] Lust J. M., Wilson P. H., Steenbergen B. (2016). Motor imagery difficulties in children with cerebral palsy: a specific or general deficit?. *Research in Developmental Disabilities*.

[49] Molina M., Kudlinski C., Guilbert J., Spruijt S., Steenbergen B., Jouen F. (2015). Motor imagery for walking: a comparison between cerebral palsy adolescents with hemiplegia and diplegia. *Research in Developmental Disabilities*.

[50] Fontes P. L. B., Moura R., Haase V. G. (2014). Evaluation of body representation in children with hemiplegic cerebral palsy: toward the development of a neuropsychological test battery. *Psychology & Neuroscience*.

[51] Fontes P. L. B., Cruz T. K. F., Souto D. O., Moura R., Haase V. G. (2016). Body representation in children with hemiplegic cerebral palsy. *Child Neuropsychology*.

[52] Steenbergen B., Craje C., Nilsen D. M., Gordon A. M. (2009). Motor imagery training in hemiplegic cerebral palsy: a potentially useful therapeutic tool for rehabilitation. *Developmental Medicine and Child Neurology*.

[53] Blanke O., Ionta S., Fornari E., Mohr C., Maeder P. (2010). Mental imagery for full and upper human bodies: common right hemisphere activations and distinct extrastriate activations. *Brain Topography*.

[54] Mohr C., Blanke O., Brugger P. (2006). Perceptual aberrations impair mental own-body transformations. *Behavioral Neuroscience*.

[55] Crescentini C., Fabbro F., Urgesi C. (2014). Mental spatial transformations of objects and bodies: different developmental trajectories in children from 7 to 11 years of age. *Developmental Psychology*.

[56] Zacks J. M., Ollinger J. M., Sheridan M. A., Tversky B. (2002). A parametric study of mental spatial transformations of bodies. *NeuroImage*.

[57] Zacks J., Rypma B., Gabrieli J. D. E., Tversky B., Glover G. H. (1999). Imagined transformations of bodies: an fMRI investigation. *Neuropsychologia*.

[58] Minnebusch D. A., Keune P. M., Suchan B., Daum I. (2010). Gradual inversion affects the processing of human body shapes. *NeuroImage*.

[59] Montirosso R., Casini E., Borgatti R., Urgesi C. (2016). Relationship between maternal sensitivity during early interaction and maternal ability in perceiving infants’ body and face. *Infancy*.

[60] Reed C. L., Stone V. E., Bozova S., Tanaka J. (2003). The body-inversion effect. *Psychological Science*.

[61] Urgesi C., Fornasari L., Canalaz F. (2014). Impaired configural body processing in anorexia nervosa: evidence from the body inversion effect. *British Journal of Psychology*.

[62] Robbins R. A., Coltheart M. (2012). The effects of inversion and familiarity on face versus body cues to person recognition. *Journal of Experimental Psychology: Human Perception and Performance*.

[63] Butti N., Montirosso R., Borgatti R., Urgesi C. (2018). Maternal sensitivity is associated with configural processing of infant’s cues in preterm and full-term mothers. *Early Human Development*.

[64] Schiltz C., Rossion B. (2006). Faces are represented holistically in the human occipito-temporal cortex. *NeuroImage*.

[65] Bauser D. A. S., Suchan B., Daum I. (2011). Differences between perception of human faces and body shapes: evidence from the composite illusion. *Vision Research*.

[66] Willems S., Vrancken L., Germeys F., Verfaillie K. (2014). Holistic processing of human body postures: evidence from the composite effect. *Frontiers in Psychology*.

[67] Robbins R. A., Coltheart M. (2012). Left-right holistic integration of human bodies. *The Quarterly Journal of Experimental Psychology*.

[68] Maurer D., Le Grand R., Mondloch C. J. (2002). The many faces of configural processing. *Trends in Cognitive Sciences*.

[69] Reed C. L., Stone V. E., Grubb J. D., McGoldrick J. E. (2006). Turning configural processing upside down: part and whole body postures. *Journal of Experimental Psychology: Human Perception and Performance*.

[70] Tanaka J. W., Gordon I. (2012). Features, configuration, and holistic face processing. *Oxford Handbook of Face Perception*.

[71] Picozzi M., Cassia V. M., Turati C., Vescovo E. (2009). The effect of inversion on 3- to 5-year-olds’ recognition of face and nonface visual objects. *Journal of Experimental Child Psychology*.

[72] Proietti V., Pisacane A., Macchi Cassia V. (2013). Natural experience modulates the processing of older adult faces in young adults and 3-year-old children. *PLoS One*.

[73] Brandman T., Yovel G. (2016). Bodies are represented as wholes rather than their sum of parts in the occipital-temporal cortex. *Cerebral Cortex*.

[74] Urgesi C., Calvo-Merino B., Haggard P., Aglioti S. M. (2007). Transcranial magnetic stimulation reveals two cortical pathways for visual body processing. *The Journal of Neuroscience*.

[75] Aviezer H., Trope Y., Todorov A. (2012). Body cues, not facial expressions, discriminate between intense positive and negative emotions. *Science*.

[76] Harris A., Vyas D. B., Reed C. L. (2016). Holistic processing for bodies and body parts: new evidence from stereoscopic depth manipulations. *Psychonomic Bulletin & Review*.

[77] Soria Bauser D. A., Schriewer E., Suchan B. (2015). Dissociation between the behavioural and electrophysiological effects of the face and body composite illusions. *British Journal of Psychology*.

[78] Vrancken L., Germeys F., Verfaillie K. (2017). Holistic integration of gaze cues in visual face and body perception: evidence from the composite design. *Journal of Vision*.

[79] Rossion B. (2013). The composite face illusion: a whole window into our understanding of holistic face perception. *Visual Cognition*.

[80] Urgesi C., Fornasari L., De Faccio S. (2011). Body schema and self-representation in patients with bulimia nervosa. *The International Journal of Eating Disorders*.

[81] van den Stock J., de Jong S. J., Hodiamont P. P. G., de Gelder B. (2011). Perceiving emotions from bodily expressions and multisensory integration of emotion cues in schizophrenia. *Social Neuroscience*.

[82] Reed C. L., Beall P. M., Stone V. E., Kopelioff L., Pulham D. J., Hepburn S. L. (2007). Brief report: perception of body posture - what individuals with autism spectrum disorder might be missing. *Journal of Autism and Developmental Disorders*.

[83] Righart R., de Gelder B. (2007). Impaired face and body perception in developmental prosopagnosia. *Proceedings of the National Academy of Sciences of the United States of America*.

[84] Busigny T., Joubert S., Felician O., Ceccaldi M., Rossion B. (2010). Holistic perception of the individual face is specific and necessary: evidence from an extensive case study of acquired prosopagnosia. *Neuropsychologia*.

[85] Candini M., Farinelli M., Ferri F. (2016). Implicit and explicit routes to recognize the own body: evidence from brain damaged patients. *Frontiers in Human Neuroscience*.

[86] Sirigu A., Grafman J., Bressler K., Sunderland T. (1991). Multiple representations contribute to body knowledge processing: evidence from a case of autotopagnosia. *Brain*.

[87] Courbois Y., Coello Y., Bouchart I. (2004). Mental imagery abilities in adolescents with spastic diplegic cerebral palsy. *Journal of Intellectual & Developmental Disability*.

[88] Reed C. L., Nyberg A. A., Grubb J. D. (2012). Contributions of visual and embodied expertise to body perception. *Perception*.

[89] DSM-5 Task Force (2013). *Diagnostic and Statistical Manual of Mental Disorders, Fifth Edition*.

[90] McDowell B. (2008). The gross motor function classification system - expanded and revised. *Developmental Medicine & Child Neurology*.

[91] Dandona L., Dandona R. (2006). Revision of visual impairment definitions in the International Statistical Classification of Diseases. *BMC Medicine*.

[92] Ospina L. H. (2009). Cortical visual impairment. *Pediatrics in Review*.

[93] Palisano R., Rosenbaum P., Walter S., Russell D., Wood E., Galuppi B. (1997). Development and reliability of a system to classify gross motor function in children with cerebral palsy. *Developmental Medicine and Child Neurology*.

[94] Wechsler D. (2003). *WISC-IV Administration Manual*.

[95] Johnston M. V., Hagberg H. (2007). Sex and the pathogenesis of cerebral palsy. *Developmental Medicine and Child Neurology*.

[96] Reiss A. L., Kesler S. R., Vohr B. (2004). Sex differences in cerebral volumes of 8-year-olds born preterm. *The Journal of Pediatrics*.

[97] Ashwal S., Russman B. S., Blasco P. A. (2004). Practice parameter: diagnostic assessment of the child with cerebral palsy: report of the Quality Standards Subcommittee of the American Academy of Neurology and the Practice Committee of the Child Neurology Society. *Neurology*.

[98] Corti C., Poggi G., Massimino M., Bardoni A., Borgatti R., Urgesi C. (2018). Visual perception and spatial transformation of the body in children and adolescents with brain tumor. *Neuropsychologia*.

[99] Korkman M., Kirk U., Kemp S. (2007). *Design and purpose of the NEPSY-II, The NEPSY*.

[100] Urgesi C., Campanella F., Fabbro F. (2011). *NEPSY-II, Contributo alla Taratura Italiana*.

[101] Richler J. J., Gauthier I. (2013). When intuition fails to align with data: a reply to Rossion (2013). *Visual Cognition*.

[102] Minnebusch D. A., Suchan B., Daum I. (2009). Losing your head: behavioral and electrophysiological effects of body inversion. *Journal of Cognitive Neuroscience*.

[103] Yovel G., Pelc T., Lubetzky I. (2010). It’s all in your head: why is the body inversion effect abolished for headless bodies?. *Journal of Experimental Psychology: Human Perception and Performance*.

[104] Green S. B., Salkind N. J. (2008). *Using SPSS for Window and Macintosh: Analyzing and Understanding Data, North*.

[105] Butson M. L., Hyde C., Steenbergen B., Williams J. (2014). Assessing motor imagery using the hand rotation task: does performance change across childhood?. *Human Movement Science*.

[106] Williams J., Reid S. M., Reddihough D. S., Anderson V. (2011). Motor imagery ability in children with congenital hemiplegia: effect of lesion side and functional level. *Research in Developmental Disabilities*.

[107] McAvinue L. P., Robertson I. H. (2007). Relationship between visual and motor imagery. *Perceptual and Motor Skills*.

[108] Pearson J., Naselaris T., Holmes E. A., Kosslyn S. M. (2015). Mental imagery: functional mechanisms and clinical applications. *Trends in Cognitive Sciences*.

[109] Avanzino L., Gueugneau N., Bisio A., Ruggeri P., Papaxanthis C., Bove M. (2015). Motor cortical plasticity induced by motor learning through mental practice. *Frontiers in Behavioral Neuroscience*.

[110] Lorey B., Bischoff M., Pilgramm S., Stark R., Munzert J., Zentgraf K. (2009). The embodied nature of motor imagery: the influence of posture and perspective. *Experimental Brain Research*.

[111] Kessler K., Thomson L. A. (2010). The embodied nature of spatial perspective taking: embodied transformation versus sensorimotor interference. *Cognition*.

[112] Minnebusch D. A., Daum I. (2009). Neuropsychological mechanisms of visual face and body perception. *Neuroscience and Biobehavioral Reviews*.

[113] Soria Bauser D., Suchan B. (2015). Is the whole the sum of its parts? Configural processing of headless bodies in the right fusiform gyrus. *Behavioural Brain Research*.

[114] Heck A., Chroust A., White H., Jubran R., Bhatt R. S. (2018). Development of body emotion perception in infancy: from discrimination to recognition. *Infant Behavior & Development*.

[115] de Heering A., Houthuys S., Rossion B. (2007). Holistic face processing is mature at 4 years of age: evidence from the composite face effect. *Journal of Experimental Child Psychology*.

[116] Crookes K., McKone E. (2009). Early maturity of face recognition: no childhood development of holistic processing, novel face encoding, or face-space. *Cognition*.

[117] Cassia V. M., Picozzi M., Kuefner D., Bricolo E., Turati C. (2009). Holistic processing for faces and cars in preschool-aged children and adults: evidence from the composite effect. *Developmental Science*.

[118] Richler J. J., Palmeri T. J., Gauthier I. (2012). Meanings, mechanisms, and measures of holistic processing. *Frontiers in Psychology*.

[119] Richler J. J., Tanaka J. W., Brown D. D., Gauthier I. (2008). Why does selective attention to parts fail in face processing?. *Journal of Experimental Psychology. Learning, Memory, and Cognition*.

[120] Richler J. J., Mack M. L., Gauthier I., Palmeri T. J. (2009). Holistic processing of faces happens at a glance. *Vision Research*.

[121] Ross D. A., Richler J. J., Gauthier I. (2015). Reliability of composite-task measurements of holistic face processing. *Behavior Research Methods*.

[122] Kim N. Y., Lee S. M., Erlendsdottir M. C., McCarthy G. (2014). Discriminable spatial patterns of activation for faces and bodies in the fusiform gyrus. *Frontiers in Human Neuroscience*.

[123] Urgesi C., Moro V., Candidi M., Aglioti S. M. (2006). Mapping implied body actions in the human motor system. *The Journal of Neuroscience*.

[124] Urgesi C., Candidi M., Ionta S., Aglioti S. M. (2007). Representation of body identity and body actions in extrastriate body area and ventral premotor cortex. *Nature Neuroscience*.

[125] Caillies S., Hody A., Calmus A. (2012). Theory of mind and irony comprehension in children with cerebral palsy. *Research in Developmental Disabilities*.

[126] Li X., Wang K., Wu J. (2014). The link between impaired theory of mind and executive function in children with cerebral palsy. *Research in Developmental Disabilities*.

[127] Sandberg A. D., Dahlgren S. (2012). Theory of mind in children with cerebral palsy: the impact of limited expressive linguistic abilities. *Access to Language and Cognitive Development*.

[128] Seth A. K. (2013). Interoceptive inference, emotion, and the embodied self. *Trends in Cognitive Sciences*.

[129] Seth A. K., Friston K. J. (2016). Active interoceptive inference and the emotional brain. *Philosophical Transactions of the Royal Society B: Biological Sciences*.

[130] Ondobaka S., Kilner J., Friston K. (2017). The role of interoceptive inference in theory of mind. *Brain and Cognition*.

[131] de Gelder B., de Borst A. W., Watson R. (2015). The perception of emotion in body expressions. *Wiley Interdisciplinary Reviews: Cognitive Science*.

[132] Frith C. D., Frith U. (2007). Social cognition in humans. *Current Biology*.

[133] Conson M., Mazzarella E., Esposito D. (2015). “Put myself into your place”: embodied simulation and perspective taking in autism spectrum disorders. *Autism Research*.

[134] Vander Heyden K. M., Huizinga M., Raijmakers M. E. J., Jolles J. (2017). Children’s representations of another person’s spatial perspective: different strategies for different viewpoints?. *Journal of Experimental Child Psychology*.

[135] Vander Heyden K. M., Huizinga M., Kan K. J., Jolles J. (2016). A developmental perspective on spatial reasoning: dissociating object transformation from viewer transformation ability. *Cognitive Development*.

[136] Richler J. J., Gauthier I. (2014). A meta-analysis and review of holistic face processing. *Psychological Bulletin*.

[137] Mondloch C. J., Pathman T., Maurer D., Le Grand R., de Schonen S. (2007). The composite face effect in six-year-old children: evidence of adult-like holistic face processing. *Visual Cognition*.

